# Evaluation of COVID-19 ECHO training program for healthcare workers in India - A Mixed-Method Study

**DOI:** 10.1186/s12913-022-08288-5

**Published:** 2022-07-08

**Authors:** Rajmohan Panda, Nivedita Mishra, Supriya Lahoti, Rajath R Prabhu, Arti Mishra, Kalpana Singh, Kumud Rai

**Affiliations:** 1 Jodhpur School of Public Health, Pal link road, Jodhpur, India; 2 Extension for Community Healthcare Outcomes (ECHO) India, Okhla phase III, New Delhi, India; 3 Hamad Medical Corporation, Doha, Qatar

**Keywords:** COVID-19, ECHO Telementoring, Capacity building, Healthcare workers, Mixed-method study

## Abstract

**Background:**

The Coronavirus Disease 2019 (COVID-19) has severely challenged healthcare delivery systems worldwide. Healthcare Workers were unable to assess and manage the cases due to limited knowledge of treating the virus and inadequate infrastructure. Digital interventions played a crucial role in the training of healthcare workers to get through the pandemic. Project Extension for Community Healthcare Outcomes (ECHO) initiated the COVID-ECHO telementoring program for strengthening the knowledge and skills of healthcare workers. The study aimed at assessing the effects of the ECHO telementoring model in the capacity building of healthcare workers in the context of COVID-19 in India.

**Method:**

We adopted a mixed-method approach with a parallel combination design. A quantitative survey was used to measure changes in the knowledge and self-efficacy among doctors and nurses. In-depth Interviews were used for qualitative exploration of perceptions and experiences of all the study participants. Student t-test and ANOVA were used to assess significant differences between mean scores across participant characteristics for different themes. Statistical significance was set at *p* < 0.05. In-depth Interviews were analyzed using Framework Analysis. The evaluation followed the first five levels of Moore’s model.

**Results:**

The results highlighted the strengthening of knowledge and skills of healthcare workers in the assessment and management of COVID-19 after the ECHO training. Learning and performance ratings were high as 96% reported an increase in knowledge and 98% were able to apply it in their clinical practices. The key challenges identified were technical issues like internet connectivity and lack of interaction due to limited visual connection. The hybrid sessions, use of video camera, feedback mechanism, and inclusion of Continuing Medical Education were recommended by participants to improve the model.

**Conclusions:**

The findings of this study are an important addition to the pre-existing literature supporting the replicability of the ECHO model in the upskilling of healthcare professionals working in underserved and remote areas, not only in the context of COVID-19 but also in other public health domains. To enhance the effectiveness of this ECHO model, the study findings may be used to refine the model and improve the areas of concern.

**Supplementary Information:**

The online version contains supplementary material available at 10.1186/s12913-022-08288-5.

## Background

The novel coronavirus disease (COVID-19) outbreak due to severe acute respiratory syndrome coronavirus 2 (SARS-CoV-2) was first reported in Wuhan, China, on 31 December 2019. Due to an alarming rise in the spread, World Health Organization (WHO) officially declared COVID-19 as a pandemic on 11 March 2020 [1]. The abrupt rise in COVID-19 cases affected the healthcare system of many countries which were unable to cope with the rising number of cases and limited knowledge of treating the novel virus. This was true especially in low- and middle- income countries (LMICs) like India where the health system is overburdened and has inadequate infrastructure [2, 3]. Previous studies suggest that the disease outbreak significantly elevated the psychological stress levels of healthcare workers (HCWs) due to rapidly changing medical information, overwhelming infection control procedures, dealing with logistic challenges, and associated stigma that leads to an increase in disease transmission and lower level of patient care [3–5]. Education intervention at the organization level can play a pivotal role in overcoming the adverse psychosocial impact caused by COVID-19 [6–8].

The training and capacity building of healthcare workers (HCWs) has been identified as one of the important strategies under the National Patient Safety Implementation Framework (NPSIF) 2018–2025 as well as in the Regional Strategy for Patient Safety (RSPS) in the WHO south-east Asia Region (2016–2025) [9]. Learning through continuous medical education and intensive training will increase the knowledge and skills to cope with public health emergencies [10]. The Government of India launched a special digital platform iGOT for training and capacity building of healthcare staff including doctors, nurses, paramedics, hygiene workers, and technicians thus stepping up efforts to fight the coronavirus pandemic [11].

Digital interventions can provide many opportunities for strengthening health systems and can be vital resources in the current public health emergency [2]. The applications of digital technology for training and capacity building during public health emergencies are well known. Digitalization of services provides an excellent forum for national and international experts to give advice and support to inexperienced HCWs through online training and mentoring [12, 13].

The Extension for Community Healthcare Outcomes (ECHO) is a model that provides informative training and case discussion by medical experts via telementoring, in urban and rural settings, connecting through the web-based videoconferencing platform. It uses a “hub and spoke” model, in which the ‘hub’ is the central physical location from which a specialist team hosts the clinic and the ‘spokes’ are HCWs who connect remotely from their workplace [14, 15]. In February 2020, Project ECHO took the initiative to implement the COVID-ECHO model for training and mentoring HCWs in India. The telementoring COVID-ECHO program is aimed at building capacity and strengthening knowledge of HCWs.

The present study aimed at assessing the effects of the ECHO telementoring model in the capacity building of HCWs in the context of COVID-19 in India. Specific objectives of the study were to:

1. Understand the barriers and facilitators in using the ECHO telementoring model for COVID-19.
2. Examine the healthcare providers’ knowledge, competence, performance, and satisfaction associated with the use of the COVID-ECHO telementoring program.
3. Explore the potential of the ECHO telementoring model for the expansion of clinical services beyond COVID-19.

## Methods

### Study design

The study involves a mixed-method approach using a parallel combination design [16], where quantitative and qualitative data were collected in the same phase. We adapted this approach to maximize the validity, reliability, and confirmability of the findings.

### Study setting and participants

The study was conducted at four ECHO hubs located in different states of India - Punjab, Assam, Kerala, and Tamil Nadu from January 2021 till April 2021. Inclusion criteria for selection of hubs was based on the minimum number of sessions (ten training sessions) conducted and the minimum number of participants (500 participants) trained. The study participants for survey data were doctors and nurses (trainees) who had received training. For qualitative data, we included other stakeholders involved in the implementation of the training program i.e. hub-leaders and trainers.

### Sampling method and recruitment

A non-probability, purposive sampling was used to select the universe of the trainees. This was followed by simple random sampling for the quantitative survey. Doctors and nurses who attended at least three or more sessions of this program and agreed to participate in the study were approached for the survey.

For qualitative inquiry, we used In-depth Interviews (IDIs). Purposive sampling with maximum variation was used to generate different perspectives from all stakeholders. Interviews were conducted until data saturation was achieved.

### Data collection and study tools

The data was collected through telephonic (cell phone) interviews to avoid exposure/risk to both data collectors and participants because of the COVID-19 situation. A data collection team that had expertise in telephonic interviews was selected and trained by the research team in a two-day training program to collect the data.

For the quantitative study, a structured survey tool was developed for this study. This questionnaire was designed keeping Moore’s Expanded Outcomes Framework for assessing learners and evaluating instructional activities. This tool was developed by adapting instruments used by previous researchers to assess similar ECHO educational projects in low- and middle-income countries [17–19]. We used a 5-point Likert scale to assess the participant ratings of knowledge and self-efficacy after attending the COVID-ECHO program. This was designed in the CS Pro software (version 7.5) – an offline electronic data capture system (EDC) and data were collected using CS Pro smartphone application.

Qualitative data was led by two researchers (NM and SL) who worked closely with the field data collectors. Separate discussion guides were developed for conducting IDIs with different stakeholders. The interviews were audio-recorded after taking participant's consent, each of which lasted for 20-30 minutes. Audio recordings were translated and transcribed verbatim by a professional transcriptionist.

### Study size

To estimate the sample number for the survey, we assumed a 10% improvement in knowledge, learning, and skills; anticipated 8% precision and response rate 60% with a design effect factor of 1.5. The sample estimated was 135 participants from each hub (state). The final sample size for this study was 540 participants. These were selected from 4 hubs that consented to participate in the study.

A total of 28 IDIs were conducted with trainees as well as other stakeholders (hub-leaders, trainers).

### Data analysis

Discrete variables are reported as frequency, proportions, and continuous variables as mean± SD. The mean scores were calculated using Likert 5 scale for the different themes. Student t-test and ANOVA were used to assess significant differences between mean scores across participant characteristics for different themes. Subgroups analysis was performed for different subgroups (doctors and nurses). The regression diagnostic and residual plot were used for testing violation of assumptions. Polynomial Linear Regression was performed to identify the factors associated with favourable learning and competence, performance, and satisfaction scores. Age and years of experience variables were used as the polynomial variables in the regression model. All statistical analysis was done using STATA Version 16 software (StataCorp. 2019. Stata Statistical Software: Release 16. College Station, TX: StataCorp LLC). All tests were two-sided, and statistical significance was set at *p* < 0.05.

The qualitative data was analysed by a framework analysis approach [20]. To familiarize with the data two researchers (NM and SL) reviewed the transcripts, coded them independently, and identified initial themes. Data coding was done using both deductive and inductive approaches. A third trained researcher (AM) helped develop consensus around themes and addressed disagreements between the two initial coders. After reconciliation, 90% agreement with the codes was achieved and the final codebook was revised. Clusters of linked codes were grouped into categories, emergent themes, and verbatim quotes. Atlas ti software version 8 was used for the data analysis.

### Data quality assurance

To check the data credibility in quantitative data, 10% of the data was analyzed by two researchers (KS and RP). The researchers conducted a preliminary descriptive analysis of the 10% data and checked for inconsistencies and missing data. To ensure the accuracy of qualitative data, we reviewed the audio recordings and compared them with transcriptions, and field notes of data collectors.

## Results

The quantitative and qualitative findings were arranged with Moore’s first five levels of evaluation. In addition, the qualitative findings explored challenges faced during the training as well as understanding the potential and scope of the viability of the ECHO program.

### Moore’s evaluation

Moore’s Model of Outcomes Assessment framework for continuing medical education (CME) focusses on the first five outcome levels - participation, satisfaction, learning, competence, and performance [21].

### Moore’s level 1 - participation

Table 1 presents the participants’ demographic characteristics. The survey included 523 participants, out of which 306 (58.5%) were doctors and 217 (41.5%) were nurses. Most of the participants were female (66%), and were nurses (92%). The overall mean age of the participants was 36.1±10.1 years. Among doctors and nurses the mean age was 36.8±10.5 years and 35.2±9.37 years, respectively.

**Table 1:** Participants demographic characteristics

Characteristics	Doctors	Nurses	Total
**N**	306	217	523
Age, mean (SD)^a^	36.9 (10.6)	35.2 (9.4)	36.2 (10.1)
Gender			
Male	161 (52.6%)	18 (8.3%)	179 (34.2%)
Female	145 (47.4%)	199 (91.7%)	344 (65.8%)
Education Qualification			
MBBS	202 (66.2%)	-	202 (38.7%)
MD	88 (28.9%)	-	88 (16.9%)
BDS	15 (4.9%)	-	15 (2.9%)
Diploma-Nursing	-	135 (62.2%)	135 (25.9%)
BSc-Nursing	-	71 (32.7%)	71 (13.6%)
MSc-Nursing	-	11 (5.1%)	11 (2.1%)
Caste			
General	171 (59.4%)	120 (55.6%)	291 (57.7%)
SC/ST	39 (13.5%)	35 (16.2%)	74 (14.7%)
OBC	78 (27.1%)	61 (28.2%)	139 (27.6%)
Site of Practice			
Primary	107 (35.7%)	107 (49.5%)	214 (41.5%)
Secondary	29 (9.7%)	31 (14.4%)	60 (11.6%)
Tertiary	143 (47.7%)	55 (25.5%)	198 (38.4%)
Private	17 (5.7%)	19 (8.8%)	36 (7.0%)
Others	4 (1.3%)	4 (1.9%)	8 (1.6%)
Location of Practice			
Rural	136 (44.9%)	115 (53.0%)	251 (48.3%)
Urban	167 (55.1%)	102 (47.0%)	269 (51.7%)
Work Experience, mean (SD)^a^	8.1 (8.8)	7.5 (8.2)	7.8 (8.5)
Source of ECHO sessions			
Internet/ECHO Website	174 (57.4%)	65 (30.0%)	239 (46.0%)
Colleagues	47 (15.5%)	61 (28.1%)	108 (20.8%)
Social Media	32 (10.6%)	13 (6.0%)	45 (8.7%)
Others	50 (16.5%)	78 (35.9%)	128 (24.6%)

^a^In completed years

Most of the participant’s (41%) practiced in primary care facilities that included primary health care centre and sub-centres. The doctors (47%) in our study were from tertiary care facilities whereas half of the nurses (49%) were from primary care facilities. One-third of the doctors (39%) were having a MBBS degree while one-fourth of nurses (26%) were having a diploma nursing degree.

### Moore’s level 2 – satisfaction

**Fig. 1:**
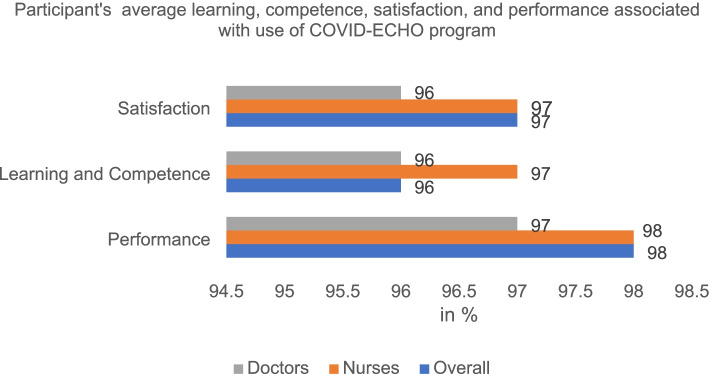
Participant's average learning, competence, satisfaction, and performance associated with the use of COVID-ECHO program

Seven questions assessed participants’ satisfaction with the COVID-ECHO telementoring program. Most participants (97%) were satisfied with the ECHO model (Figure 1), with participants either ‘agreeing’ or ‘strongly agreeing’ with the majority of satisfaction items. They felt that the sessions were easy to understand and have met their expectations in terms of information shared and duration of session. The t-test and ANOVA results showed that the dentists (doctors with BDS degree) were more likely to be satisfied (32.9±3.8) with the session compared to doctors with MBBS degree (30.2±3.3) or MD degree (31.3±3.4; *p*=0.002). The doctors who were practicing in private facilities showed greater satisfaction levels (32.9±3.0) when compared to doctors practicing at primary (29.8±3.5), secondary (31.4±3.3), tertiary (31.0±3.3; *p*=0.002) facilities (Table 2).

**Table 2:** T-test and ANOVA summary—Doctors

	***N* = 306**	**Learning & Competence**		**Performance**		**Satisfaction**	
**Variables**		**Mean (SD)**	***p*-value**	**Mean (SD)**	***p*-value**	**Mean (SD)**	***p*-value**
**Gender**			0.096		0.12		0.15
Male	161	52.3 (5.4)		17.4 (1.9)		30.4 (3.2)	
Female	145	53.4 (5.9)		17.8 (2.1)		31.0 (3.6)	
**Highest Qualification**			**0.001**		** < 0.001**		**0.002**
MBBS	202	52.2 (5.6)		17.3 (1.9)		30.3 (3.3)	
MD	88	53.7 (5.5)		18.0 (2.0)		31.3 (3.4)	
BDS	15	57.2 (4.8)		18.9 (2.0)		32.9 (3.8)	
**Caste**			0.11		0.087		0.083
General	171	53.7 (5.5)		17.9 (2.0)		31.2 (3.3)	
SC/ST	39	52.5 (6.0)		17.6 (2.1)		30.4 (3.4)	
OBC & Others	78	52.1 (5.8)		17.3 (2.0)		30.2 (3.7)	
**Practice Site**			**0.006**		**0.040**		**0.002**
Primary facility	107	51.3 (5.5)		17.2 (1.9)		29.8 (3.5)	
Secondary facility	29	53.9 (5.9)		17.8 (2.1)		31.4 (3.3)	
Tertiary facility	143	53.7 (5.5)		17.8 (2.0)		31.0 (3.3)	
Private facility	17	54.6 (6.0)		18.4 (2.2)		32.9 (3.0)	
**Practice Location**			**0.014**		**0.007**		**0.002**
Rural	136	52.0 (5.9)		17.3 (2.0)		30.0 (3.4)	
Urban	167	53.6 (5.4)		17.9 (2.0)		31.3 (3.3)	

The qualitative findings complement the quantitative findings and suggest that participants were satisfied with the model. The findings indicate that the sessions were interactive and participants’ experience with trainers was satisfactory.

“The model was very good, it was very practical, and it met my expectations” (Doctor, ID 5.20)

“It was a good interaction in the sessions. we got to meet people sitting thousands of kilometres away, (and with) doctors across the globe” (Trainer, ID 17.3)

Participants also noted the advantages of the virtual learning platform.

“It's [medical profession] dynamic, things keep changing, New protocols or new guidelines come, we get to learn and do things now and then. So, this platform helps us to gain knowledge faster and easily” (Doctor, ID 21.16)

“We need to go someplace to learn [for physical sessions]. For example, if I need to learn something. I need to travel someplace. Once we got it virtually, there is no need to travel, and it is a time-saving factor” (Doctor. ID 22.10)

### Moore’s level 3 - learning

The learning and competence among the participants were assessed together in the quantitative survey. Around 96% of participants reported improvement in knowledge and clinical skills for assessing and recognizing COVID-19 symptoms after attending the program (Figure 1). Participants reported increased confidence in advising and managing patients with COVID-19 after attending these sessions

The t-test and ANOVA results showed that the dentists (doctors with BDS degree) (57.2 ±4.8) had a higher mean score on the knowledge scale as compared to doctors with MBBS (52.2±5.6) or MD degrees (53.7±5.5; *p*=0.001). Doctors practicing in private health facilities which include personal clinics reported higher mean score on the knowledge scale (54.6 ±6.0) when compared with doctors practicing in primary (51.3±5.5), secondary (53.9±5.9), and tertiary (53.7±5.5; *p*=0.006) facilities (Table 2). Among the nurses, there was no significant difference in the mean scores on the knowledge scale related to their qualification, practice site, and practice location (Table 3). The findings from the qualitative data are in tune with the quantitative findings.

“I liked the fact that when we started off, we had no knowledge about the COVID. But through these sessions, we got a lot of knowledge” (Nurses, ID 14.3)

**Table 3:** T-test and ANOVA summary – Nurses

	***N* = 217**	**Learning & Competence**		**Performance**		**Satisfaction**	
**Variables**		**Mean (SD)**	***p*-value**	**Mean (SD)**	***p*-value**	**Mean (SD)**	***p*-value**
**Gender**							
Male	18	43.9 (4.2)	0.89	17.7 (1.9)	0.64	30.4 (2.5)	0.64
Female	199	43.8 (4.2)		17.5 (1.7)		30.8 (3.2)	
**Highest Qualification**							
Diploma Nursing	135	44.0 (4.3)	0.74	17.4 (1.6)	0.82	30.8 (3.1)	0.64
BSc. Nursing	71	43.6 (3.9)		17.6 (1.8)		30.9 (3.4)	
MSc. Nursing	11	43.1 (5.7)		17.4 (2.0)		29.9 (2.9)	
**Caste**							
General	120	43.7 (3.9)	0.17	17.4 (1.5)	0.34	30.7 (3.1)	0.64
SC/ST	35	45.0 (4.7)		17.9 (1.8)		31.3 (3.5)	
OBC & Others	61	43.4 (4.5)		17.4 (1.9)		30.7 (3.0)	
**Practice Site**							
Primary facility	107	44.0 (4.3)	0.48	17.6 (1.7)	0.29	31.0 (3.2)	0.22
Secondary facility	31	44.5 (3.5)		17.7 (1.5)		31.1 (3.0)	
Tertiary facility	55	43.6 (4.8)		17.4 (1.9)		30.7 (3.3)	
Private facility	19	42.6 (3.1)		16.8 (1.5)		29.2 (2.5)	
**Practice Location**							
Rural	115	44.3 (4.3)	0.093	17.6 (1.7)	0.48	31.2 (3.2)	**0.048**
Urban	102	43.3 (4.1)		17.4 (1.7)		30.3 (3.1)	

A few study participants mentioned improved task performance and skill development.

“On this ECHO platform, we trained them on how to wear PPE (Personal Protective Equipment) kits, how to wash hands, how to protect healthcare workers” (Trainer, ID 15.5)

“So, dealing with dementia cases is also very new to us. It was helpful interacting with people from all over the country with experts from NIMHANS (National Institute of Mental Health and Neurosciences)” (Doctor, ID 5.27)

### Moore’s level 4 - competence

The qualitative findings show that the training helped participants gain confidence in their practice.

“Some of the people have sent their messages to me that after listening to you on this particular topic I could manage with this thing” (Trainer, ID 3.4)

“It was really helpful in gaining enough confidence to write on the prescription of psychiatric drugs” (Doctor, ID 5.14)

### Moore’s level 5 - performance

Participants were also asked to rate their self-reported performance on a 5-point Likert scale. Majority of participants (above 95%) have reported that they have implemented the learning in their clinical practice and disseminated the knowledge with colleagues (Figure 1).

The dentists (doctors with BDS degree) showed a higher self-reported performance mean score (18.9±2.0) compared to doctors with MBBS degree (17.3±1.9) or a MD degree (18.0±2.0; *p*=<0.001). The doctors practicing in urban areas had a higher performance mean score (17.9±2.0) compared to doctors in rural areas (17.3±2.0; *p*=0.007) (Table 2). There were no statistically significant differences in performance mean scores among nurses related to their qualification, practice site, and practice location (Table 3). The participants reported that they had applied these knowledge and skills in treating their patients. Many of the trainees spoke of gaining knowledge and felt that they were more efficient in handling patients after joining the training program.

“I used it [learning] in ICU, and in the wards on my patients” (Doctor, ID 12.10) “As far as I feel, my patients benefitted too.” (Nurse, ID 13.18)

Participants also felt that there was a positive change in their behaviour towards patient care after attending the training program.

“My empathy level has increased that I can see in my interaction with the patients. Earlier I was a little bit robotic I have now become more human. Now I am looking into the psychosocial aspects also an equally important part, it is having a tremendous impact in recovery of the patient.” (Hub-leader, ID 4.13)

Regression analysis: After adjusting for demographic factors i.e. age, gender, qualification, caste, years of experience, practice site, and polynomial terms of age and years of experience in current job, the dentists (doctors with BDS degree) had a significantly higher learning score (Coef. 3.97 95% CI :0.89,7.04; *p*=0.012) compared to doctors with MBBS degree. The doctors practicing in secondary (Coef. 2.76 95% CI :0.37,5.14; *p*=0.024) and tertiary (Coef. 2.11 95% CI :0.41,3.80; *p*=0.015) care settings had significantly higher learning scores as compared to primary care settings. The doctors practicing in secondary care settings (Coef. 1.53 95% CI :0.08,2.98; *p*= 0.03) were more satisfied as compared to doctors in primary care settings. The dentists had a significantly higher self-reported performance score (Coef. 1.28 95% CI :0.19,2.37; *p*=0.021) as compared to MBBS doctors (Additional file 3, Appendix 1).

### Challenges during COVID-ECHO training

Five themes (limited interaction, difficulty to follow up, time constraint, technical issues, and need for physical training) were identified as challenges faced by the participants during these training. A summary of challenges is mentioned in (Table 4).

**Table 4:** Summary of challenges

Theme	**Description**	**Quotes**
Limited interaction	Sessions were less interactive as compared to face-to-face sessions	*“The interaction was more of audio. We could not interact since videos were not on all the time” (Hub leader, ID 9.21)*
Difficult to follow-up	Follow up with the participants to assess the practical implications of the training program is difficult	*“We could not do the quality check by follow up, in the way that how much they have learned and how much they have applied” (Hub leader, ID 9.12)*
Time constraint	Session’s time conflicts with their duty hours	*“We have some barriers, first one is the time. Most of them [COVID-ECHO sessions] are on-duty time” (Hub leader, ID 7.10)*
Technical issues	Internet connectivity is the biggest limitation of the program	*“Sometimes the host network wasn’t that good. So, they would get automatically disconnected, then they will have to reconnect, we have to wait” (Doctor, ID 21.5)*
Need for physical training	Face-to-face training is appropriate for gaining skills in the clinical domain	*“Hands-on experience on patients, hands-on CPR, isn't it? These are the things that you cannot teach online. These are the things for which you have to be assisted by your trainers” (Trainer, ID 17.6)*

### Viability of ECHO program

The majority of participants felt that the training program is effective, and most of the trainees were in the favor of attending future ECHO sessions.

“I liked their manner of explaining very much, their method of telling. That is why I would certainly want to attend their next session” (Nurse, ID 13.23)

“So, beyond COVID-19 and cancer there is the scope of expanding this ECHO session [training in other clinical services]” (Trainer, ID 10.24)

Based on their experience, participants also recommended strategies to overcome the challenges and improve the model. We summarized the recommendations under the given themes in (Table 5).

**Table 5:** Recommended strategies to improve model

Theme	**Description**	**Quotes**
Timing of sessions	Duration of 30 to 60 min, preferably post-lunch was considered appropriate	*“According to me, perfect time was half an hour or one hour” (Nurse, ID 14.7)* *“We have OPD, but post-lunch is less, so ideally post-lunch is the ideal time I would say” (Doctor, ID 10.5)*
Interactive sessions	Compulsory use of video cameras by participants and offering recorded sessions	*“If the videos were on and could see each other. It would improve interaction in certain ways (Hub leader, ID 9.26)* *“If these sessions can be recorded so that we get to watch them later on, it will be better” (Nurse, ID 13.10)*
Hybrid sessions	Hybrid model blending both synchronous and asynchronous models of training	*“We can combine like a hybrid session where you have some degree of remote learning coupled with the physical part. That will again act like a good platform for us” (Trainer, ID 1.5)*
Publicity of program	Publicizing the ECHO platform through various media sources	*“It should be posted on YouTube” (Doctor, ID 8.16)*
Feedback mechanism	Strengthen the existing feedback mechanism and ensure the collection of responses from the participants	*“We had a portal…feedback was given in the written form. When we asked for the feedback form so very few people had given the feedback form.” (Hub leader, ID 9.35)*
Inclusion of CME	CME accreditation for the ECHO program can enhance participation as well as help in better branding of the program	*“A program like EHCO if it could get CME Credit, I think more people will also participate and that money also which they invested will go in a better place” (Doctor, ID 5.15)*

## Discussion

Our study findings reveal that participants of the COVID-ECHO telementoring program were satisfied with the virtual ECHO platform, the sessions as well as the trainers conducting the sessions. Participants reported improvement in their knowledge and confidence in COVID-19 assessment and management after attending the sessions. These findings are similar to other studies that have used Project ECHO for building capacity in chronic pain [22], hypertension [23], and pain management in dementia [24]. Participants also expressed that the COVID- ECHO sessions improved their clinical practice and benefitted their patients. The results of this study indicate the ability of the COVID-ECHO program to reach out to remote populations for building capacity in evidence-based COVID-19 protocols. This was made possible through telementoring and an interactive system that integrates telecommunications into the planning, design, and delivery of training programs.

Interprofessional collaborative practice is considered an ideal method of improving staff satisfaction while benefitting the community [25, 26]. Participants perceived that virtual training was more relevant than physical training for collaboration because of the advantages of a larger and more diverse audience. The discussions in the COVID sessions were centered around expert’s opinions and global best practices. Our quantitative and qualitative results are consistent with the findings of previous assessments of Project ECHO [27–29].

Our study findings regarding providers’ level of satisfaction towards the telementoring program, our data is aligned with previous reports by Zhou et al. [29]. The review by Zhou examined thirteen studies that measured satisfaction levels in primary care providers. In all the thirteen studies conducted in North America, the participants indicated a high level of satisfaction and performance with the ECHO telementoring model [27, 29]. Our findings regarding participants’ improvement in their interaction as well as practice with patients after attending the ECHO sessions are in accordance with other studies which report improved relationships with patients after attending the ECHO training [30, 31]. The mean ratings for the learning, competence, satisfaction, and performance in this study were found to be consistently above 4 on the 5-point scale. This finding is similar to the other studies that have also assessed the effectiveness of the ECHO telementoring model in North America and Ireland from 2000 to 2018 [14, 19].

Participants also described some of the limitations of the training such as administrative issues like inconvenient schedule of sessions, and technical issues like internet connectivity, and video conferencing. Technical issues, in particular internet connectivity and bandwidth, have been identified as problematic by other studies evaluating online training [24, 32]. By their very nature, virtual learning environments and platforms like ECHO are subject to technical issues, such as security, network, and low bandwidth. Hence, the strategies for addressing them on a priority basis will help build more confidence in such models. A further challenge in the training as stated by few participants was a lack of interaction due to limited visual connection and face-to-face interactivity with the trainees. A few participants believe skill development cannot be achieved through online training while some other participants do not see these issues as major problems in future ECHO clinics.

To improve the model few suggestions were put forward by the participants. One of them was to have ECHO sessions not only in the clinical domain but also in other areas like training human resources and strengthening community awareness in the area of COVID-19. The potential of ECHO sessions to go beyond the clinical domain to cover vulnerable populations has been found effective in a study conducted in the USA [33]. To improve active participation and effective interaction, participants emphasized the use of visual interactions in addition to audio ones. To strengthen the model, a hybrid session of remote learning coupled with face-to-face training would be useful. Participants also felt the inclusion of certification and continuing medical education credits will expand the training program.

The COVID-19 pandemic has shown how lack of knowledge among HCWs has been a major barrier for managing outbreaks. Training programs like ECHO has the potential to reach and strengthen the healthcare workforce in collaboration with public health universities and government institutions [34, 35]. The results of this study are an important addition to the pre-existing literature supporting the replicability of the Project ECHO model in the upskilling of health professionals and capacity building of healthcare providers working in underserved and remote areas. The findings of this study indicate that the ECHO tele-mentoring model has strong potential to be replicated in low-middle-income settings like India. The current training was an excellent example of collaboration of ECHO India with academic medical centres, departments of health in several states, and non-profit organizations. Such models are critical and serve as disruptive innovators for complex problems plaguing the delivery of specialized care to underserved populations [34].

### Strengths and limitations

This study is one of the first to use a mixed-method approach to assess an online model for building the capacity of healthcare providers in the context of the COVID-19 in India. Despite current COVID wave constraints, we were able to collect good quality data from different stakeholders across different states in India. The limitations of the study include the smaller number of qualitative interviews with nurses due to constraints posed by the second wave of COVID. We were unable to validate the quantitative survey tool due to resource constraints. The retrospective design of the study involved data collection after 6 months of the COVID- ECHO training and there could be a potential recall bias by study participants.

## Conclusion

Digital health innovations are rapidly changing the delivery of healthcare services globally. The results of our study support that the ECHO telementoring model has the potential to bring this transformation through building capacities in HCWs working across different geographies. This model can bridge the knowledge asymmetry and help build capacity in healthcare providers working in inaccessible communities. The findings of this study may be used to improve the model. This will help expand and enhance the effectiveness of the ECHO model.

## Supplementary Information

**Additional file 1.**

**Additional file 2.**

**Additional file 3.**

**Additional file 4.**

## Data Availability

All data generated or analysed during this study are included in this published article [as supplementary information files].

## Abbreviations

COVID-19: Coronavirus disease 2019
SARS-CoV-2: Severe Acute Respiratory Syndrome Coronavirus 2
WHO: World Health Organization
HCWs: Healthcare Workers
LMICs: Low- and Middle-Income Countries
NPSIF: National Patient Safety Implementation Framework
RSPS: Regional Strategy for Patient Safety
ECHO: Extension for Community Healthcare Outcomes
IDIs: In-depth interviews
EDC: Electronic Data Capture
IEC: Institutional Ethics Committee
PIS: Participant Information Sheet
JSPH: Jodhpur School of Public Health
CPR: Cardio-Pulmonary Resuscitation
OPD: Outpatient Department
CME: Continuing Medical Education
