# Supplementary material for: Evaluation of COVID-19 ECHO training program for healthcare workers in India - A Mixed-Method Study
Source: BMC Health Serv Res. 2022 Jul 8;22:883. doi: 10.1186/s12913-022-08288-5 (PMC9264289; doi:10.1186/s12913-022-08288-5)
Supplement: Supplementary file 1 — Additional file 1. [file 12913_2022_8288_MOESM1_ESM.docx]

**Participant Information Sheet for Trainees**

**Study title:** Evaluation of effectiveness of COVID-19 training of health care workers in India: A mixed method study of the ECHO tele mentoring model

**PI Name:** Dr Rajmohan Panda

**PI Contact address:** Jodhpur School of Public Health (JSPH)

**JSPH-IEC approval number and date:**

**Why are we doing this study?**

The coronavirus disease 2019 (COVID-19) global pandemic has severely challenged healthcare delivery systems worldwide, disrupted traditional modalities of education at all levels and altered norms of social interaction everywhere. The novelty of this virus has created a need for training of healthcare workers to handle the outbreak. In the current scenario, technologies are playing a crucial role in keeping our society functional in a time of lockdowns and quarantines. Therefore, ECHO-India provided training to the health care providers for COVID-19 clinical management in Maharashtra via tele-ECHO sessions and you are one of them. Therefore, with this evaluation we would like to identify barriers and facilitators that you faced, and asses the effectiveness in the capacity building of health care providers in context of COVID-19 emergency.

**Who is doing this study?**

Jodhpur School of Public Health (JSPH) is doing the study in collaboration with ECHO India.

**Why am I being asked to take part in this study?**

You are being asked to take part in this study because you are one of the key stakeholders of the ECHO tele-mentoring program. We request you to consider participating as we feel that you will be able to provide us with information that will help in understanding what influenced participant engagement; how participants valued the ECHO experience; and what the utility of the ECHO tele-mentoring experience has been for participants such as yourself. If you agree, I would like to ask you about ECHO tele-mentoring program and request you to answer the questions to the best of your knowledge.

**Do I have to take part in this study?**

Your participation in this study is purely voluntary. It is your choice whether to participate or not. You do not have to take part in this study if you do not wish to do so, and choosing to participate will not affect your work in any way.

If you do take part, it will take about 45 minutes including going through the Participant Information Sheet (PIS) and consent form. We want to assure you that the information that you provide will be kept confidential. Your name or other information that could identify you will not appear in any record or report. You are free to withdraw from this study at any time should you change your mind.

**What information will be collected from me and how? OR What will happen to me if I take part in this study?**

You will be asked to participate in an audio call according to your convenience on telephonic or online platforms. The information sought from you purely pertains to your experience and perception about ECHO tele-mentoring program. Few questions will also need your personal Information, apart from information required in the survey /and or interview. We will ensure confidentiality of the survey/and or the interview. Irrespective of what you answer, there will not be any consequences of the same.

**Will interview be audio-recorded?**

Yes, this information will be audio recorded with your permission. We will ensure confidentiality of this recording. The information that you give is confidential and will not be shared by any one in any manner that can identify you. The audio of the interview will be saved in the Jodhpur School of Public Health (JSPH) repository for 3 years. We assure you that the information so collected from this study will be kept privately in a secure manner and none of your personal details will appear on this information.

**What are the possible disadvantages/risks of taking part?**

We are asking you to share with us some information related to your work and responsibility at the institution and you may feel uncomfortable talking about some of the topics. You do not have to answer any question you do not want to answer.  Nor do you need to take part in the interview if you don’t wish to do so, and that is all fine. You do not have to give us any reason for not responding to any question or for refusing to take part in the interview. The information that you give is confidential and will not be shared by any one in any manner that can identify you.

**How will I benefit from this study? OR What are the possible benefits of taking part?**

By participating in the study, you are contributing to the understanding of barriers and facilitators for ECHO tele-mentoring program and its effectiveness in light of COVID-19. This will have benefit to state leaders and service providers who are responsible for developing best practices to tackle COVID-19 and individuals affected by COVID-19

**How will privacy and confidentiality of my information be maintained?**

We will ensure your privacy is maintained during the interviews. We will be collecting personal information however all the information that you share with us will be coded and we will not share the information you provide with anyone other than the research team. The information so collected from this study will be kept privately in a secure manner and none of your personal details will appear on this information. The information provided by you hence will not be attributed to you by name. The knowledge that we get from this study will be shared with you and the decision makers in a workshop before it is made widely available to the public. It is possible that your de-identified data i.e. data after removing your name, address and other identifying information may be shared with other researchers in the future.

**What is the system for data safety and storage?**

Data will be maintained by Jodhpur School of Public Health (JSPH) for at least seven years after completion of the project or as specified by the funding agency. Appropriate scanned copy of paper and electronic storage mechanisms would be made so that data can be accessed as required. Data will not be destroyed or removed without prior approval of the Research Management Committee.

**Will I get any compensation or reimbursement for participating?** (In terms of time, travel, man-days lost from work, etc.)

No compensation will be provided to you for participating in this study.

**Will I get any compensation for injury, if any caused due to my participation in this study?**

There is no active intervention in the project. We do not anticipate that you will incur any injury due to your participation in this study.

**Can I leave the study? What will happen to information collected from me?**

You do not have to take part in this study if you do not wish to do so. You may stop participating in the interview at any time that you wish. In case you decide to withdraw from the study, all information collected from you will be destroyed.

**Will I be informed about the results of this study?**

Yes, the final report will be shared with Program managers, policy makers and service providers

**Will I be given a copy of this PIS?**

Yes, you will be provided with a copy of this participant information sheet to keep with you. You are free to discuss this with any one you wish to consult.

**Who can I contact for additional information?**

If you have additional questions about the study at any point in time, please contact the study staff or PI at Jodhpur School of Public Health (JSPH).

| **PI contact information**  Dr Rajmohan Panda  Independent consultant and Adjunct Professor  Health System and Policy Research  Jodhpur School of Public Health (JSPH) | **JSPH-IEC contact information** |
| --- | --- |

**CONSENT FORM**

**Study title:** Evaluation of effectiveness of COVID-19 training of health care workers in India: A mixed method study of the ECHO tele mentoring model

**PI Name:** Dr Rajmohan Panda

**PI Contact address:** Jodhpur School of Public Health (JSPH)

**JSPH-IEC approval number and date:**

*“I have read the foregoing information, or it has been read to me. The purpose of this study has been explained to me in my own language. I have had the opportunity to ask questions about it and any questions I have asked have been answered to my satisfaction. I have received a copy of the participant information sheet and consent form”.*

**Please tick the box**

| 1 | I have been informed by the investigators about the process including the nature, objective and known likely risks and benefits related to this study and I have understood them. |  |
| --- | --- | --- |
| 2 | I understand that my participation is voluntary and that I am free to withdraw at any time, without giving any reason and without it affecting me in any way. |  |
| 3 | I understand that any information I provide will be confidential and no identifiable personal data will be published. |  |
| 4 | I also understand that if I have any concerns regarding this research, I can contact the PI or the IEC member secretary. |  |
| 5 | By signing this form, I give my free and informed consent to take part in this study as outlined in the information sheet and this consent form. |  |
| 6 | I understand that audio recording will be done of the interview. I agree for my recording to be presented in academic presentations or reports. |  |
| 7 | I understand that there is a chance that my information may be shared with other researchers in the future. I understand that this information will not include my name, or any personal identification. |  |

**_____________________________________ __________________________________**

**Name of Participant Signature**

**Date: ______________________________**

**____________________________________ ___________________________________**

**Name of Person Obtaining Consent Signature**

**Date: ______________________________**
