# Supplementary material for: Evaluation of COVID-19 ECHO training program for healthcare workers in India - A Mixed-Method Study
Source: BMC Health Serv Res. 2022 Jul 8;22:883. doi: 10.1186/s12913-022-08288-5 (PMC9264289; doi:10.1186/s12913-022-08288-5)
Supplement: Supplementary file 2 — Additional file 2. [file 12913_2022_8288_MOESM2_ESM.docx]

**Survey questionnaire for the trainees (Doctors and Nurses)**

**Participant Identification Number (PID) :**

| **Section I: Identification Details** | | |
| --- | --- | --- |
| **S.No.** | **Question** | **Response** |
| 1.1 | Name of Hub |  |
| 1.2 | Name of the study participant |  |
| 1.3 | Contact number |  |
| 1.4 | Place of residence | City/village ____________ District ___________ State ___________ |
| 1.5 | Sector *(Please tick)* | Rural  / Urban  / Semi-Urban |
| 1.6 | Name of interviewer |  |
| 1.7 | Date of assessment | ____ / ____ / ________ *(dd/mm/yyyy)* |
| 1.8 | What is your profession? | Doctor  Nurse |

| **Section II: Socio-Demographic Characteristics** | | | | |
| --- | --- | --- | --- | --- |
| **S.No.** | **Question** | **Response(s)** | **Code** | **Remark**  **(No response=99)** |
| 2.1 | What is your age? | ___________ (Age in completed years) | | |
| 2.2 | What is your gender? | Male | 1 |  |
|  |  | Female | 2 |  |
|  |  | Transgender | 3 |  |
| 2.3 | What is your highest qualification? | MBBS | 1 |  |
|  |  | MD | 2 |  |
|  |  | AYUSH Practioner | 3 |  |
|  |  | BDS | 4 |  |
|  |  | Diploma-Nursing | 5 |  |
|  |  | B.Sc - Nursing | 6 |  |
|  |  | M.Sc- Nursing | 7 |  |
|  |  | Others (Please specify)  ________________________ | 9 |  |
| 2.4 | What is your religion? | Hindu | 1 |  |
|  |  | Muslim | 2 |  |
|  |  | Sikh | 3 |  |
|  |  | Christian | 4 |  |
|  |  | Others (Please specify)  ________________________ | 9 |  |
| 2.5 | What is your caste category? | General | 1 |  |
|  |  | SC | 2 |  |
|  |  | ST | 3 |  |
|  |  | OBC | 4 |  |
|  |  | Others (specify)  _________________________ | 9 |  |
| 2.6 | How many years have you been in your current job? | ________________ (in completed years) | | |
| 2.7 | What is the current site of your practice? | Sub-Center (SC) | 1 |  |
|  |  | Primary Health Center (PHC) | 2 |  |
|  |  | Community Health Center (CHC) | 3 |  |
|  |  | Health and Wellness Center (HWC) | 4 |  |
|  |  | District or Sub-district hospital (DH/SDH) | 5 |  |
|  |  | Others (Please specify)  _________________________ | 9 |  |
| 2.8 | What is your location of practice? | Rural | 1 |  |
|  |  | Urban | 2 |  |
|  |  | Semi-urban | 3 |  |
|  |  | Other  -------------------- | 9 |  |
| 2.9 | How many ECHO sessions have you attended? | ________________ (in number) | | |
| 2.10 | From where did you got to know about these ECHO sessions? | Internet / ECHO site | 1 |  |
|  |  | Newspaper | 2 |  |
|  |  | Colleagues | 3 |  |
|  |  | Social media | 4 |  |
|  |  | Others (Please specify)  __________________________ | 9 |  |

| **Section III: Information on ECHO COVID-19 Sessions** | | | | | | |
| --- | --- | --- | --- | --- | --- | --- |
| **Response** | | Strongly disagree | Disagree | Neither agree nor disagree | Agree | Strongly agree |
| **Code** | | 1 | 2 | 3 | 4 | 5 |
| **Learning and self-confidence evaluation statements** | | | | | | |
| 3.1 | After the sessions, did you gain new knowledge applicable for your work? |  |  |  |  |  |
| 3.2 | After the sessions, did you gain new clinical skills applicable for your work? |  |  |  |  |  |
| 3.3 | After the sessions, did you feel confident in your clinical knowledge of the disease? |  |  |  |  |  |
| 3.4 | After the sessions, did you feel confident in assessing and recognizing symptoms of COVID? |  |  |  |  |  |
| 3.5 | After the sessions, did you feel confident in interpreting laboratory values associated with COVID? |  |  |  |  |  |
| 3.6 | After the sessions, did you feel confident in establishing a diagnosis for COVID?  (Applicable only for Doctors) |  |  |  |  |  |
| 3.7 | After the sessions, did you feel confident in prescribing medicines and treatment for the patients?  (Applicable only for Doctors) |  |  |  |  |  |
| 3.8 | After the sessions, did you feel confident assessing treatment response in patients with COVID?  (Applicable only for Doctors) |  |  |  |  |  |
| 3.9 | After the sessions, did you feel confident that you are using best-practice approaches for management of COVID?  (Applicable only for Doctors) |  |  |  |  |  |
| 3.10 | After the sessions, did you feel confident in reporting symptoms of COVID  (Applicable only for Nurses) |  |  |  |  |  |
| 3.11 | After the sessions, did you feel confident in administering medications/ drugs to COVID patients  (Applicable only for Nurses) |  |  |  |  |  |
| 3.12 | After the sessions, did you feel confident in giving advice and managing patients with COVID? |  |  |  |  |  |
| 3.13 | After the sessions, did your ability to communicate with patients and families about COVID have improved? |  |  |  |  |  |
| 3.14 | Did the ECHO sessions have improved the way health professionals communicate with each other? |  |  |  |  |  |
| **Need and experience evaluation statements** | | | | | | |
| 3.15 | Did access to expertise on COVID-19 is needed by you and your team? |  |  |  |  |  |
| 3.16 | Did the ECHO sessions on COVID-19 give you an opportunity to ask questions on the disease? |  |  |  |  |  |
| 3.17 | Did the ECHO session on COVID-19 give you an opportunity to provide feedback on the sessions? |  |  |  |  |  |
| 3.18 | Did the sessions keep you well-informed about symptoms of COVID patients in treatment? |  |  |  |  |  |
| 3.19 | While taking part in these sessions, were you able to communicate the barriers faced by you? |  |  |  |  |  |
| 3.20 | Were the barriers addressed by the ECHO team? |  |  |  |  |  |
| **Transfer of knowledge from ECHO sessions to clinical care evaluation statements** | | | | | | |
| 3.21 | Were you able to apply the knowledge you learned from the sessions to patients in your facility? |  |  |  |  |  |
| 3.22 | Were you able to share knowledge with the staff about COVID discussed in the ECHO sessions you attended? |  |  |  |  |  |
| 3.23 | Did the learning benefit your organization? |  |  |  |  |  |
| 3.24 | Did the ECHO sessions on COVID 19 provide a unique opportunity to discuss the cases with different providers in the field? |  |  |  |  |  |
| **Satisfaction evaluation statements** | | | | | | |
| 3.25 | Did the sessions deliver the information as per your expectation? |  |  |  |  |  |
| 3.26 | Were the sessions of sufficient duration? |  |  |  |  |  |
| 3.27 | Were these ECHO sessions easy to understand? |  |  |  |  |  |
| 3.28 | Do you think that ECHO sessions should be continued for COVID? |  |  |  |  |  |
| 3.29 | Would you like to attend future ECHO sessions on COVID? |  |  |  |  |  |
| 3.30 | Are you interested in participating in future ECHO sessions on other focus areas? |  |  |  |  |  |
| 3.31 | Will you recommend these sessions to your friends and colleagues? |  |  |  |  |  |

| **Section IV: Experience with Logistic Arrangements** | | | | | | | | | | |
| --- | --- | --- | --- | --- | --- | --- | --- | --- | --- | --- |
| **S.No.** | | **Logistic Issues** | | **Responses** | | | | | **Remarks**  **(No response=99)** | |
| 4.1 | | How do you rate the overall internet connectivity for the session? | | Excellent | | | 1 | |  | |
|  |  |  |  | Good | | | 2 | |  |  |
|  |  |  |  | Fair | | | 3 | |  |  |
|  |  |  |  | Poor | | | 4 | |  |  |
| 4.2 | | How would you rate the coordination of the Hub team? | | Excellent | | | 1 | |  | |
|  |  |  |  | Good | | | 2 | |  |  |
|  |  |  |  | Fair | | | 3 | |  |  |
|  |  |  |  | Poor | | | 4 | |  |  |
| 4.3 | | How would you rate the timing scheduled for the sessions? | | Excellent | | | 1 | |  | |
|  |  |  |  | Good | | | 2 | |  |  |
|  |  |  |  | Fair | | | 3 | |  |  |
|  |  |  |  | Poor | | | 4 | |  |  |
|  |  |  |  | No | | | 0 | |  |  |
| 4.4 | | Did you get any incentive (TA/DA) to attend the program? | | Yes | | | 1 | |  | |
|  |  |  |  | No | | | 0 | |  |  |
| **Section V: Experience with Trainers/Faculties** | | | | | | | | | | |
| **Response** | | | Strongly disagree | | Disagree | Neither agree nor disagree | | Agree | | Strongly agree |
| **Code** | | | 1 | | 2 | 3 | | 4 | | 5 |
| 5.1 | Were the trainers knowledgeable in the subject? | |  | |  |  | |  | |  |
| 5.2 | Were the presentation skills of the trainer good? | |  | |  |  | |  | |  |
| 5.3 | Were the trainers interactive with the participants? | |  | |  |  | |  | |  |
| 5.4 | Was the language used by the trainer understandable? | |  | |  |  | |  | |  |
