# Supplementary material for: Evaluation of COVID-19 ECHO training program for healthcare workers in India - A Mixed-Method Study
Source: BMC Health Serv Res. 2022 Jul 8;22:883. doi: 10.1186/s12913-022-08288-5 (PMC9264289; doi:10.1186/s12913-022-08288-5)
Supplement: Supplementary file 3 — Additional file 3. [file 12913_2022_8288_MOESM3_ESM.docx]

**Appendix 1.**

Regression table – for doctors

|  | **Learning & Competence** | | **Performance** | | **Satisfaction** | |
| --- | --- | --- | --- | --- | --- | --- |
| **Variables** | **coef. (95% CI)** | **p-value** | **coef. (95% CI)** | **p-value** | **coef. (95% CI)** | **p-value** |
| Age | -0.04 (-0.62,0.53) | 0.891 | 0.01 (-0.19,0.21) | 0.923 | -0.04 (-0.38,0.31) | 0.844 |
| Age* | 0 (-0.006, 0.006) | 0.984 | 0 (-0.002, 0.001) | 0.740 | 0 (-0.003, 0.004) | 0.935 |
| Gender |  |  |  |  |  |  |
| Male | Ref |  | Ref |  | Ref |  |
| Female | 1.25 (-0.08,2.57) | 0.065 | 0.38 (-0.1,0.85) | 0.118 | 0.62 (-0.18,1.43) | 0.129 |
| Highest Qualification |  |  |  |  |  |  |
| MBBS | Ref |  | Ref |  | Ref |  |
| MD | 0.62 (-1.1,2.33) | 0.48 | 0.52 (-0.09,1.13) | 0.092 | 0.56 (-0.48,1.6) | 0.292 |
| BDS | 3.97 (0.9,7.05) | **0.012** | 1.28 (0.19,2.38) | **0.021** | 1.74 (-0.13,3.61) | 0.069 |
| Caste |  |  |  |  |  |  |
| General | Ref |  | Ref |  | Ref |  |
| SC/ST | -0.69 (-2.67,1.29) | 0.492 | -0.16 (-0.86,0.55) | 0.662 | -0.42 (-1.63,0.78) | 0.488 |
| OBC & Others | -0.83 (-2.39,0.73) | 0.295 | -0.41 (-0.96,0.14) | 0.146 | -0.53 (-1.48,0.41) | 0.269 |
| Years of experience | 0.29 (-0.03,0.6) | 0.076 | 0.05 (-0.06,0.16) | 0.371 | 0.11 (-0.09,0.3) | 0.283 |
| Years of experience* | -0.006 (-0.01, 0.002) | 0.133 | 0 (-0.003, 0.002) | 0.590 | -0.002 (-0.007, 0.002) | 0.391 |
| Practice Site |  |  |  |  |  |  |
| Primary facility | Ref |  | Ref |  | Ref |  |
| Secondary facility | 2.76 (0.37,5.14) | **0.024** | 0.68 (-0.17,1.53) | 0.114 | 1.53 (0.08,2.98) | **0.039** |
| Tertiary facility | 2.11 (0.41,3.81) | **0.015** | 0.26 (-0.34,0.86) | 0.396 | 0.72 (-0.31,1.76) | 0.169 |
| Private facility | 1.54 (-1.65,4.72) | 0.343 | 0.36 (-0.77,1.49) | 0.536 | 1.71 (-0.23,3.64) | 0.084 |
| Practice Location |  |  |  |  |  |  |
| Rural | Ref |  | Ref |  | Ref |  |
| Urban | 0.83 (-0.71,2.36) | 0.289 | 0.42 (-0.13,0.96) | 0.134 | 0.86 (-0.07,1.79) | 0.071 |

*polynomial variables

Table 2. Regression table – for nurses

|  | **Learning & Competence** | | **Performance** | | **Satisfaction** | |
| --- | --- | --- | --- | --- | --- | --- |
| **Variables** | **coef. (95% CI)** | **p-value** | **coef. (95% CI)** | **p-value** | **coef. (95% CI)** | **p-value** |
| Age | 0.29 (-0.31,0.89) | 0.344 | -0.04 (-0.28,0.2) | 0.74 | 0.28 (-0.16,0.72) | 0.213 |
| Age* | 0 (-0.01,0) | 0.421 | 0 (0,0) | 0.793 | 0 (-0.01,0) | 0.262 |
| Gender |  |  |  |  |  |  |
| Male | Ref |  | Ref |  | Ref |  |
| Female | -0.26 (-2.41,1.89) | 0.814 | -0.09 (-0.95,0.76) | 0.827 | 0.37 (-1.22,1.96) | 0.645 |
| Highest Qualification |  |  |  |  |  |  |
| Diploma Nursing | Ref |  | Ref |  | Ref |  |
| BSc Nursing | -0.47 (-1.81,0.86) | 0.485 | 0.02 (-0.51,0.56) | 0.926 | -0.14 (-1.13,0.85) | 0.78 |
| MSc Nursing | -0.96 (-3.82,1.9) | 0.507 | 0.31 (-0.83,1.44) | 0.597 | -0.77 (-2.89,1.34) | 0.471 |
| Caste |  |  |  |  |  |  |
| General | Ref |  | Ref |  | Ref |  |
| SC/ST | 1.38 (-0.34,3.1) | 0.114 | 0.43 (-0.25,1.11) | 0.215 | 0.46 (-0.81,1.74) | 0.471 |
| OBC & Others | -0.06 (-1.45,1.33) | 0.93 | 0 (-0.55,0.55) | 0.997 | 0.12 (-0.91,1.15) | 0.813 |
| Years of experience | -0.16 (-0.45,0.14) | 0.297 | 0.02 (-0.09,0.14) | 0.688 | -0.08 (-0.29,0.14) | 0.482 |
| Years of experience* | 0 (0,0.01) | 0.402 | 0 (0,0) | 0.44 | 0 (-0.01,0.01) | 0.788 |
| Practice Site |  |  |  |  |  |  |
| Primary facility | Ref |  | Ref |  | Ref |  |
| Secondary facility | 0.4 (-1.39,2.19) | 0.663 | 0 (-0.71,0.71) | 0.998 | -0.03 (-1.36,1.29) | 0.962 |
| Tertiary facility | 0.02 (-1.64,1.68) | 0.984 | -0.31 (-0.97,0.35) | 0.358 | -0.05 (-1.27,1.18) | 0.941 |
| Private facility | -0.76 (-3.01,1.48) | 0.503 | -0.88 (-1.78,0.01) | 0.053 | -1.38 (-3.04,0.28) | 0.102 |
| Practice Location |  |  |  |  |  |  |
| Rural | Ref |  | Ref |  | Ref |  |
| Urban | -0.66 (-2.04,0.71) | 0.345 | 0.03 (-0.52,0.58) | 0.915 | -0.63 (-1.65,0.39) | 0.223 |

*polynomial variables
